# Transcriptome sequencing identifies novel persistent viruses in herbicide resistant wild-grasses

**DOI:** 10.1038/srep41987

**Published:** 2017-02-06

**Authors:** Federico Sabbadin, Rachel Glover, Rebecca Stafford, Zuriñe Rozado-Aguirre, Neil Boonham, Ian Adams, Rick Mumford, Robert Edwards

**Affiliations:** 1 Centre for Novel Agricultural Products, Department of Biology, University of York, York YO10 5DD UK; 2 Fera Science Ltd., Sand Hutton, York YO41 1LZ UK; 3 School of Agriculture, Food and Rural Development, Newcastle University, NE1 7RU UK

## Abstract

Herbicide resistance in wild grasses is widespread in the UK, with non-target site resistance (NTSR) to multiple chemistries being particularly problematic in weed control. As a complex trait, NTSR is driven by complex evolutionary pressures and the growing awareness of the role of the phytobiome in plant abiotic stress tolerance, led us to sequence the transcriptomes of herbicide resistant and susceptible populations of black-grass and annual rye-grass for the presence of endophytes. Black-grass (*Alopecurus myosuroides; Am*) populations, displaying no overt disease symptoms, contained three previously undescribed viruses belonging to the *Partititiviridae* (AMPV1 and AMPV2) and *Rhabdoviridae* (AMVV1) families. These infections were widespread in UK black-grass populations and evidence was obtained for similar viruses being present in annual rye grass (*Lolium rigidum*), perennial rye-grass (*Lolium perenne*) and meadow fescue (*Festuca pratensis*). In black-grass, while no direct causative link was established linking viral infection to herbicide resistance, transcriptome sequencing showed a high incidence of infection in the NTSR Peldon population. The widespread infection of these weeds by little characterised and persistent viruses and their potential evolutionary role in enhancing plant stress tolerance mechanisms including NTSR warrants further investigation.

As our knowledge of the complexity of the microbial community associated with plants (the phytobiome) grows, there is an increasing awareness of the unexpected benefits to the host of these interactions^1^. In addition to the well-studied classic symbioses (eg: nodulation), members of the phytobiome can contribute by largely undefined mechanisms, to plant vigour, stress tolerance and disease resistance^1^,^2^. Within the scope of enhancing abiotic stress tolerance, we have recently reviewed the evidence that endophytic microbes influence herbicide tolerance, either through directly metabolizing toxic xenobiotics, or by inducing more generic plant defence mechanisms^3^. Following on from this line of enquiry, we have become interested in any potential role for the phytobiome in herbicide resistance in weed populations.

The chemical control of black-grass (*Alopecurus myosuroides*) and annual rye-grass (*Lolium rigidum*) in cereal crops is an increasing challenge to sustainable arable production, due the steady rise of herbicide resistance^4^,^5^,^6^. In the UK, herbicide resistance in black-grass has been reported on over 16000 farms and can develop through two major mechanisms^7^. Target site-based resistance (TSR), arises from point mutations in plant genes encoding proteins targeted by herbicides, rendering them insensitive to inhibition. In the case of black-grass and annual rye-grass the most important current TSR mutations arise in acetolactate synthase (ALS) and acetyl CoA carboxylase (ACCase); these lead to a reduced sensitivity to inhibition to herbicides from the sulphonylurea and aryloxyphonoxypropionate/cyclohexanedione classes respectively^7^. TSR can be counteracted by alternating the use of herbicides with different modes of action. In contrast, non-target site resistance (NTSR) results from the co-ordinated enhanced expression of multiple genes involved in xenobiotic detoxification, resulting in resistance to herbicides, irrespective of their mode of action^5^,^6^. In wild–grasses, both TSR and NTSR are selected for following repeated exposure to graminicides^8^. Whereas TSR behaves as a Mendelian trait, linked to mutations in a single gene, NTSR is inherited in a complex manner, behaving as a multigenic quantitative trait^8^. As such, the inheritance of NTSR in wild grasses is influenced by environmental factors in addition to herbicide selection^4^,^9^. With an interest in the potential role of microorganisms as an environmental driver of NTSR, we have used a non-targeted next generation sequencing approach to examine the phytobiome of black-grass and rye-grass. We have then examined the incidence of the identified endophytes in weed populations independently isolated from different sites in the UK that vary in their resistance to sulphonylurea and aryloxyphonoxypropionate/cyclohexanedione graminicides.

## Results

### Identification of persistent viruses in black-grass

RNA extracted from leaves of apparently healthy (symptomless) black-grass and annual rye-grass was sequenced using a GS-FLX (Roche). For black-grass, the wild-type sensitive (WTS) Rothamsted and NTSR Peldon populations were used, while for annual rye-grass commercially available WTS and SLR31 resistant plants were tested. The assembled contigs were analysed for the presence of non-plant sequences by comparing the sequences to the NCBI nr database using blastx v2.2.28+ 10,11,12, followed by taxonomic determination using MEGAN^13^. Based on this analysis, while no evidence for the presence of bacterial or fungal RNA were found, a large number of sequences of viral origin were readily identified with amino-acid sequence homology to viruses belonging to the *Alphapartitivirus* and *Varicosavirus* genera.

**Figure 1:**
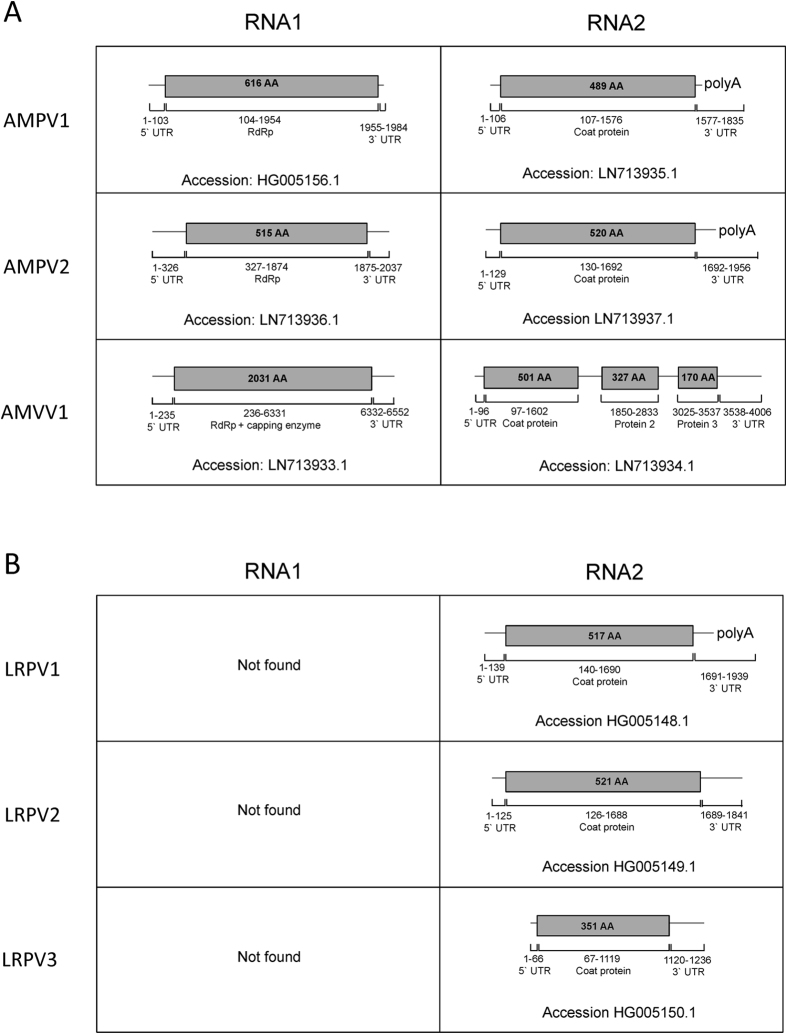
Schematic representation of the fully assembled genome sequences of the viruses found in (**A**) black-grass (AMPV1/2 & AMVV1) and (**B**) annual rye-grass (LRPV). Total nucleotide and protein length of the coded ORFs are indicated, together with the putative function and the accession number.

In black-grass, low coverage, long read data generated using the GS-FLX gave only partial sequence coverage for the new viruses and it was not obvious whether the plants were infected with a single virus from each genera, or several closely related viruses. To extend the coverage of the viral genomes, high coverage, short read datasets were produced using RNAseq (Illumina). Three replicates were taken from each of the WTS (Roth 09), NTSR (Peldon 05) and TSR (Notts 05) populations characterized previously^7^. The improved coverage, allowed the re-construction of the complete RNA genomes of the three viruses (Fig. 1A). Each genome was bipartite, comprising an RNA1, coding for an RNA-dependent RNA polymerase (RdRP) and an RNA2, coding for (at least) one coat protein. The respective RNA1 and RNA2 sequences were each paired to a single virus based on DNA sequence identity in the un-translated linker regions (UTRs).

**Figure 2:**
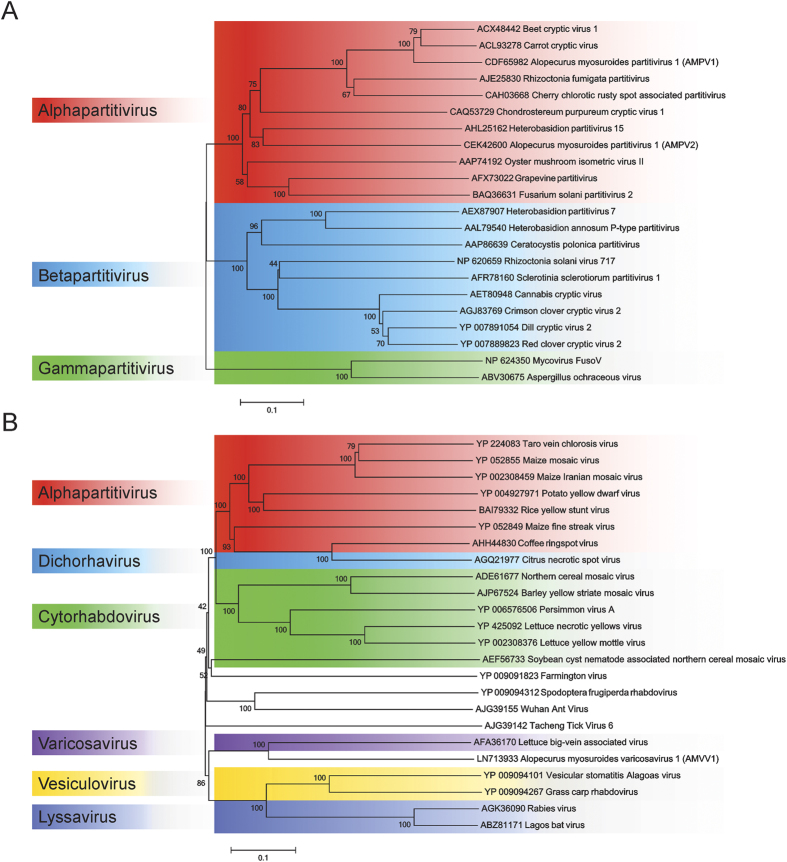
Phylogenetic analysis of the RNA1 of the three assembled black-grass persistent viruses. (**A**) Protein alignment of the RdRp demonstrates that AMPV1 and AMPV2 belong to the *Alphapartitivirus* genus within the *Partitiviridae* family. (**B**) Protein alignment of the polyprotein of AMVV1 demonstrates that it can be assigned to the *Rhabdoviridae* family, within the genus *Varicosavirus.*

The sequence of the assembled virus genomes were subjected to BlastX annotation and phylogenetic analysis based on the RNA1 sequences (Fig. 2). Of the three viruses, two were homologous to the dsRNA family of *Partitiviridae* and were called *Alopecurus myosuroides partitivirus* 1 (AMPV1) and *Alopecurus myosuroides partitivirus* 2 (AMPV2) respectively. The *Partitiviridae* sequences identified in black-grass were most closely related to members of the plant-infecting *Alphapartitivirus* genera, but were sufficiently diverse to fall into two different clades (Fig. 2).

The third viral genome found in black-grass had some protein sequence similarity to members of the *Rhabdoviridae* family, but was most similar to the *Varicosavirus Lettuce big-vein associated virus* (LBVaV) (Fig. 2), with which it shares a bi-partite genome structure^14^,^15^. The virus was called *Alopecurus myosuroides varicosavirus* 1 (AMVV1). Within the bipartite AMVV1 genome, RNA1 encoded a poly-protein made up of an RNA-dependent RNA polymerase, a capping region and a capping enzyme, while RNA2 coded for three putative coat proteins (Fig. 1A). BlastX analysis of the RNA2 of AMVV1 found low-homology protein matches to the coat protein 1 of *Lettuce big-vein associated virus* (LBVaV) and *Tobacco stunt virus* (TSV), both members of the genus *Varicosavirus*. No homology or function could be associated with the other putative protein coding regions in AMVV1, with similar ambiguous links to function determined with the orthologous proteins of LBVaV^16^.

### Identification of persistent viruses in other wild-grass species

Transcriptome sequencing was also used to identify any viral sequences present in annual rye-grass (*Lolium rigidum*) NTSR (SLR31) and WTS populations. *De novo* assembly of the transcriptome derived from long read, low coverage sequencing (GS-FLX) produced the complete RNA2 sequence of three putative members of the *Partitiviridae* (Fig. 1B). Protein alignments showed that two of these sequences (accessions: HG005148 and HG005149) shared homology with the RNA2 of AMPV2 and would likely be classified as members of the genus *Alphapartitivirus*. These viruses were tentatively named *Lolium rigidum* partitiviruses 1–3 (LRPV1, LRPV2 and LRPV3), although the absence of RNA1 for each meant the full identity and classification of these viruses was not possible (Fig. 1B).

The presence of related viral sequences in black-grass and annual rye-grass suggested that further wild grasses could harbour uncharacterised persistent viruses. The assembled genomes of the viruses found in black-grass and annual ryegrass were used to search the transcriptome and genome databases of wild grasses available on NCBI. BlastN searches against the Transcriptome Shotgun Assembly (TSA) and Expressed Sequence Tag (EST) databases using the RNA1 and RNA2 sequence of AMVV1 identified several un-annotated orthologous sequences in perennial rye-grass (*Lolium perenne)* and meadow fescue (*Festuca pratensis* = *Alopecurus pratensis*). While the sequences from *F. pratensis* were too fragmented for reassembly, the AMVV1 scaffold did allow for the reconstruction of the majority of the putative RNA1 of the related virus from *L. perenne*. Further searches revealed the full length RNA2 was already available un-annotated in public TSA databases in two versions (GAYX01053802.1; GAYX01053803.1). One of these sequences coded for a protein 2 with the same length as that predicted for AMVV1, whilst the other version had an additional 41 amino acids (data not shown).

### Occurrence and abundance of persistent viruses in wild-grass populations

After normalising the EST counts within the RNAseq libraries, it was apparent that the abundance of AMVV1sequences in black-grass was higher in NTSR compared to WTS plants (Table 1). To allow for a more extensive screen for the presence of these viral sequences in black-grass populations, oligonucleotide primers were designed for RT-PCR to amplify the viral sequences corresponding to the coat proteins of RNA2 for AMPV1, AMPV2 and AMVV1 respectively (Table 2). RNA was extracted from leaves of black-grass plants derived from 10 populations sampled from different locations in the UK (Table 3). These populations had previously been characterised as either being WTS, TSR or NTSR with respect to their resistance to herbicides^7^,^17^. To confirm their herbicide resistance phenotype, the 10 samples were tested for their susceptibility to two commercially available graminicides, Atlantis WG^®^ and Cheetah ^®^ Gold. acting on ALS and ACCase targets respectively (Table 3). In addition, each population had been assessed previously for their relative enhancement in herbicide detoxification associated with NTSR (Table 3)^7^,^17^.

As a wild out-crossing weed, black-grass plants isolated from field sites are by their nature highly heterogeneous, with individuals within herbicide-resistant populations varying in their tolerance to graminicides. Thus, within the population tested it was already known that individuals varied in their susceptibility to herbicides due to variations in the incidence of TSR mutations^7^. To test whether these populations varied in their incidence of viral infection, individual plants from six different populations were tested using RT-PCR for the presence of AMVV1, AMPV1 and AMPV2 (Table 4). These results were of interest, as they presented an opportunity to examine the frequency of viral infection in individual plants present in identifiable populations. Following virus testing, individual infected plants were found in all populations, demonstrating that these viruses are widespread in the UK (Table 4). While the presence and abundance of each of the viruses varied within each set sampled, the well described NTSR Peldon population showed a high incidence of infection with all three viruses (Table 4).

## Discussion

The results presented identify several previously undescribed persistent viruses which are widespread in black-grass populations in the UK. Related sequences were also present in other wild grasses, including annual rye-grass, which like black-grass can be a major problem weed in cereal production. The most abundant viral sequences, belonged to AMPV1 and AMPV2, members of the *Alphapartitivirus* a genus of persistent plant viruses that cause symptomless latent infections within their host. These viruses are vertically transmitted via transfer from pollen into embroyos in many crops and wild specie and are highly stable within their hosts, surviving both thermotherapy and meristem culturing^2^,^18^. Members of the wider *Partitiviridae* family are widespread in plants, fungi and protozoa and have previously been identified in a number of metagenomic studies^18^. In addition the previously undescribed virus AMVV1 was identified in black-grass. AMVV1 was tentatively assigned to the genus *Varicosavirus*, based on similarity to the type member *Lettuce big-vein associated virus* (LBVaV)^14^,^16^. While initially considered to be the causal agent of lettuce big-vein disease, evidence suggests that though LBVaV is widespread amongst its host, it is not responsible for the characteristic symptoms of the disease^16^. Similarly, AMVV1 provoked no overt disease symptom in black-grass. Although the source of AMVV1 was not investigated, LBVaV is transmitted to plants though fungal infection by members of the *Chytridiomycetes*, notably *Olpidium virulentus*^19^,^20^. *In addition to being* widespread, *O. virulentus* can persist in the soil for more than 20 years and is known to infect monocots^20^. It is therefore a plausible vector for AMVV1 infection in grass weeds. With all the viruses identified, while their respective sequences were readily amplified from cDNA prepared from total RNA, no amplification products were obtained from genomic DNA. This suggested that neither AMPV or AMVV viruses had integrated into the host plant chromatin.

While widespread in the grass weeds tested, it was not clear whether or not the hosts derived any benefit from these viral infections. There are examples of plant-infecting partitiviruses having a mutualistic relationship with the plant host. One study on salt stress in *Lolium perenne* identified evidence of a greater prevalence of a deltapartitivirus within tolerant populations^21^. In the current study, we have been interested in the relationship between the abundance of persistent viruses in black-grass and changes in abiotic stress tolerance linked to NTSR-based herbicide resistance. While the incidence of infection to all three viruses was particularly marked in the well studied Peldon NTSR population (Table 4), no further associative link between infection and NTSR could be established in black-grass. By manipulating innate defence mechanisms in the host, plant viruses are known to invoke broad ranging stress tolerance pathways in plants^22^,^23^. This protection can extend to induced herbicide resistance. Thus, the baculovirus p35 gene can suppress apoptosis in passion fruit and as a consequence, enhance tolerance to the herbicide glufosinate, by suppressing cell death caused by the secondary effects of chemical injury^24^. The basis for this virally-induced activation of host defences is poorly understood, but has been linked to the induction of protective antioxidant responses in some species^25^. As viral infection is known to enhance tolerance to abiotic stress and enhance fitness in a wide variety of plants^2^, we speculate that the presence of these viruses could provide an improved biochemical and genetic background for NTSR to evolve in wild grasses in the field. Given the widespread occurrence of NTSR in grass weeds and our lack of understanding of its functional links to stress tolerance, these findings suggest that further study of the links between herbicide resistance and infection by persistent viruses is warranted.

## Materials and Methods

### Plant analysis

Seeds from herbicide-susceptible and -resistant black-grass and annual rye-grass derived from previously described populations (Table 3) were heat treated at 30 °C for 14 d prior to use. For transcriptome studies, seeds were sown into potting medium (John Innes No. 2) and germinated in a growth room (20 °C, under a 16:8 h light: dark photoperiod). Plants were then harvested after 14 days. For all other studies, plants were sown (20 seeds per 12 cm i.d.pot) in peat-based compost and grown under glass. To determine their tolerance to herbicides, black-grass populations were sprayed with Atlantis WG^®^ and Cheetah ^®^ Gold (Bayer CropScience, UK). Spray trials were performed in triplicate, with each population of *Alopecurus myosuroides* treated with five different concentrations of the two herbicides. Atlantis WG^®^ was applied at rates equivalent to 0, 100, 400, 800 and 1600 g/Ha respectively (normal field rate = 400 g/Ha). Cheetah^®^ Gold was applied at; 0, 0.625, 2.5, 10 and 20 l/Ha (normal field rate = 1.25 l/Ha). Treatments were assembled in a randomised order and phytotoxicity assessments scored visually in comparison to formulation treatments at 7, 14 and 21 days after treatment (DAT). RNA samples for PCR and next generation sequencing were prepared from individual shoots (150 mg) in triplicate after freezing in liquid Nitrogen, homogenizing with a pestle and mortar then extracting with the RNeasy kit with on-column DNAse treatment (Qiagen, UK).

### Transcriptome sequencing (low coverage – long read)

RNA samples were used to generate double-stranded cDNA using the SMARTer^TM^ PCR cDNA Synthesis Kit and Advantage^®^ 2 PCR Kit (Clontech, Mountain View, CA). Double stranded cDNA was fractionated into smaller fragments (500–800 bp) by nebulisation. Emulsion PCR was carried out using the Lib-L-LV emPCR Kit with samples run on a GS-FLX sequencer (Roche). For sequence analysis, after quality control (QC) and removal of SMART cDNA amplification primers, each of the four datasets was subjected to *de novo* genome assembly with Newbler v2.6 (Roche/454 Life Sciences). The only deviation from default settings was the application of the [–urt] option to improve the production of contigs in low depth regions of the assembly. BlastN and BlastX searches were carried out using blast + executables v2.2.25+ against a local copy of Genbank (downloaded 30/11/2011). Blast results were visualised with MEGAN v4.63^13^ with the following LCA parameters: Minimum support = 1, minimum score = 50, top percent = 10, win score = 0 and minimum complexity = 0. Confirmation of protein coding regions in selected contigs was carried out using MetaGeneMark with default settings^26^. Data from each of the four samples was passed through a basic quality check with all sequences with an average quality (phred) score of <20 and length <40 bp discarded. Multiplex identifiers (MIDs) and SMART cDNA amplification primers were then identified and removed. Genomic datasets were assembled in preference to cDNA to improve the quantity and quality of non-host contigs.

### Transcriptome sequencing (high coverage – short read)

Total RNA (4 μg) was used to produce a poly-A enriched, indexed, TruSeq V2 Illumina sequencing library following the manufacturer’s instructions. Libraries were quantified using Qubit hs-double stranded DNA kits (Invitrogen, UK). They were then pooled in equimolar quantities and purified using Ampure XP beads (Truseq manual), prior to checking for size and quality using a Tapestation (Agilent). The two resulting libraries were sequenced on two lanes of a HiSeq sequencer (Illumina).

After an initial QC step to trim poor quality regions with a Phred quality score of <25 and remove reads <50 bp, the remaining 302254761 sequences were assembled with the transcriptome assembler Trinity using default settings^27^. A total of 180206 components (genes) were assembled, with isoforms raising the total number of contigs to 373555. Non-host derived contigs were identified using Blastx to search the viral genome fragments identified from the GS-FLX data and further verified using a Blastx search against the NCBI nr database. Alignment of the amino acid sequences of the viral RNA-dependent RNA polymerases was performed with ClustalW within the MEGA6 package^28^, prior to the construction of neighbour-joining trees with 1000 bootstrap replicates.

### Reverse transcriptase-PCR (RT-PCR)

All RNA extracts were tested using primers targeting the coat protein region of the RNA2 of AMVV1, AMPV1 and AMPV2 (Table 2). Total RNA (1 μl) was added to a 24 μl reaction containing Verso™ 1-Step RT-PCR ReddyMix™ Kit (Thermo Scientific) and 400 mM of each primer. The assays were performed in a Bio-Rad C1000^TM^ thermal cycler (Bio-Rad laboratories) using the cycling conditions,48 °C for 30 mins for the reverse transcription step, 98 °C for 2 mins, followed by 35 cycles of 98 °C for 10 s, 63 °C for 30 s and 72 °C for 1 min and a final step of 72 °C for 5 min. Amplification products were separated on a 1.2% agarose gel stained with ethidium bromide and visualised on a UV transilluminator.

## Additional Information

**How to cite this article**: Sabbadin, F. *et al*. Transcriptome sequencing identifies novel persistent viruses in herbicide resistant wild-grasses. *Sci. Rep.* **7**, 41987; doi: 10.1038/srep41987 (2017).

**Publisher's note:** Springer Nature remains neutral with regard to jurisdictional claims in published maps and institutional affiliations.

## Figures and Tables

**Table 1:** Relative abundance of virus reads as a percentage of total reads following transcriptome analysis (using Illumina) for three populations of black-grass (Peldon 05, Notts 05 and Roth 09).

Sample/replicate	Total number of reads	Relative abundance of virus reads (%)		
		AMPV1	AMPV2	AMVV1
Peldon 05/1	13556109	0.084	0.154	0.035
Peldon 05/2	14405457	0.190	0.053	0.055
Peldon 05/3	14674	0.190	0.204	0.075
Notts 05/1	7625139	0.421	0.116	0
Notts 05/2	6362601	0.091	0.039	3.143 × 10^−5^
Notts 05/3	10446490	0.084	0.111	4.786 × 10^−5^
Roth 09/1	4609793	0.146	0.065	4.338 × 10^−5^
Roth 09/2	3876546	0.231	0.089	5.159 × 10^−5^
Roth 09/3	8917820	0.048	0.005	2.242 × 10^−5^

**Table 2:** Primer sequences designed to RNA 2 of AMVV1, AMPV1 and AMPV2.

Virus (accession number)	Primer	Sequence (5′-3′)
AMVV1 (LN713934)	AMVV1 F	TACCTGACTCTGACAACTCAAAGGAGCCAGG
AMVV1 (LN713934)	AMVV1 R	TAGTCCTGTGCCAGAGTCCACTGCTTAGTTC
AMPV1 (LN713935)	AMPV1 F	ACGCCACTGAACAATTCACTGGCTC
AMPV1 (LN713935)	AMPV1 R	TTGAGCCGACGAAGAAGCGACTGTAC
AMPV2 (LN713937)	AMPV2 F	TCACCCGCTTTGGATACTATTGGGTTGC
AMPV2 (LN713937)	AMPV2 R	ATCAAAGCCTATGATGGGGCTCTGTGACTCTAG

**Table 3:** Results following testing of black-grass population for susceptibility to herbicides Atlantis and Cheetah.

Population	County	Location	ALS Mutations (%)	ACCase Mutation (%)	NTSR (Enhanced Metabolism)	Lethal Dose ATLANTIS (400 g/Ha) % damage 21 DAT	Lethal Dose CHEETAH (1.25 l/Ha) % damage 21 DAT
Roth 09	Hertfordshire	Broadbalk	0	0	L	85	100
LongC 08	Oxfordshire	Chalgrove	42	8.5	M	28	15
Notts 05	Nottinghamshire	Notts	0	22.5	L	81	6
Kent1 02	Kent	Kent-Survey	0	0	H	75	13
Hor 08	Oxfordshire	Oxford	50	25	—	16	28
Suffolk 09	Suffolk	Suffolk survey	0	27.5	H	33	17
Peldon 05	Essex	Peld02 Sulfolk survey	50	0	H	25	17
Velc 08	Lincolnshire	Velcourt	32.5	31.5	—	25	10
Warren 09	Bedfordshire	Conts Atl G/Ha60	31.5	18.75	M	49	21
R30 08	Cambridgeshire	Huntingdon	45	27.5	—	25	15

Further details of these populations and the identification of the mutations in their target site proteins are as referenced^7^,^17^. NTSR (enhanced metabolism refers to the relative rates of herbicide detoxification reported as H = high, M = medium, L = Low, — not reported).

**Table 4:** Results of RT-PCR testing of individual plants for AMVV1, AMPV1 and AMPV1 from RNA extracted from 6 black-grass populations.

Population	Numbers of infected plants/plants tested		
	AMVV1	AMPV1	AMPV2
Peldon 05	21/36	23/36	16/36
Cambridgeshire 08	0/5	4/5	3/5
Peldon 07	5/5	4/5	3/5
Notts 05	0/5	2/5	2/5
Roth 09	0/5	0/5	2/5
Kent1 02	0/5	2/5	1/5

Extracts were tested for the Cp region of RNA2 for each virus.
